# Multiple Techno-Functional Characteristics of *Leuconostoc* and Their Potential in Sourdough Fermentations

**DOI:** 10.3390/microorganisms9081633

**Published:** 2021-07-30

**Authors:** Denise C. Müller, Sandra Mischler, Regine Schönlechner, Susanne Miescher Schwenninger

**Affiliations:** 1 Food Biotechnology Research Group, Institute of Food and Beverage Innovation, Zurich University of Applied Sciences (ZHAW), 8820 Wädenswil, Switzerland; denise.mueller@zhaw.ch (D.C.M.); sandra.mischler@zhaw.ch (S.M.); 2 Department of Food Science and Technology, University of Natural Resources and Life Sciences (BOKU), 1190 Vienna, Austria; regine.schoenlechner@boku.ac.at

**Keywords:** *Leuconostoc*, non-conventional starter cultures, sourdough fermentations, exopolysaccharides, mannitol, antifungal

## Abstract

In this study, the potential of *Leuconostoc* as non-conventional sourdough starter cultures was investigated. A screening for antifungal activities of 99 lactic acid bacteria (LAB) strains revealed high suppression of bakery-relevant moulds in nine strains of *Leuconostoc* with activities against *Penicillium* sp., *Aspergillus* sp., and *Cladosporium* sp. Mannitol production was determined in 49 *Leuconostoc* strains with >30 g/L mannitol in fructose (50 g/L)-enriched MRS. Further, exopolysaccharides (EPS) production was qualitatively determined on sucrose (40 g/L)-enriched MRS agar and revealed 59 EPS positive *Leuconostoc* strains that harboured dextransucrase genes, as confirmed by PCR. Four multifunctional *Lc. citreum* strains (DCM49, DCM65, MA079, and MA113) were finally applied in lab-scale sourdough fermentations (30 °C, 24 h). *Lc. citreum* was confirmed by MALDI-TOF MS up to 9 log CFU/g and pH dropped to 4.0 and TTA increased to 12.4. Antifungal compounds such as acetic acid, phenyllactic and hydroxyphenyllactic acids were determined up to 1.7 mg/g, 2.1 µg/g, and 1.3 µg/g, respectively, mannitol up to 8.6 mg/g, and EPS up to 0.62 g/100 g. Due to the observed multifunctionalities and the competitiveness in the natural flour microbiota present in sourdoughs, non-conventional LAB genera such as *Leuconostoc* seem promising for application in sourdough-based bakery products.

## 1. Introduction

Based on metabolic activities, the application of a lactic acid bacteria (LAB) fermentation is used broadly in the food industry, e.g., for traditional sourdough fermentation [1]. LAB mainly belonging to species of the former genus of *Lactobacillus* that were recently classified by Zheng et al. [2] into the novel genera *Lactiplantibacillus (Lpb.) plantarum* subsp. *plantarum* (former: *Lactobacillus* (*Lb.*) *plantarum*), *Levilactobacillus (Lev.) brevis* (former: *Lb. brevis*), and *Fructilactobacillus (F.) sanfranciscensis* (former: *Lb. sanfranciscensis*) dominate the sourdough microbiota [3]. The application and suitability of these LAB genera as starter cultures in sourdough fermentations has been researched for years [3,4]. However, non-conventional LAB genera such as *Leuconostoc* (*Lc.*) and *Weissella* (*W*.) are still less studied but bear high potential as starter cultures in sourdough fermentations due to their adaptation and performance in sourdough fermentations [5].

Various functional activities of LAB have been researched in the last decades. LAB derived mannitol mainly produced by heterofermentative LAB [6] such as *Lc. citreum* can be used as sugar substitute that is relevant for the baking industries in the production of sugar-reduced baked goods [7]. Further, the formation of microbial exopolysaccharides (EPS) as a natural texture enhancing agent has been studied by Chen et al. [8] and was extensively studied in, e.g., *Leuconostoc*, *Weissella*, and species of the former genus of *Lactobacillus* such as *Lpb. plantarum* subsp*. plantarum* (former: *Lb. plantarum*) or *Limosilactobacillus (Li.) reuteri* (former *Lb. reuteri*) [9,10,11,12,13]. EPS synthetizing genes were investigated in *Lc. citreum* FDR241 by Coda et al. [14] and microbial built dextran, a glucan consisting of α(1→6)-linked glucopyranosyl units, was reported for bread texture-affecting properties [8,11].

A relevant aspect industrial bakeries have to face is fungal spoilage of the products due to fungal spores on surfaces, equipment, production staff, or aerocontamination on the production site [15]. Antifungal metabolites in LAB were studied extensively, focusing mainly on *Lb. amylovorus* [16,17], *Li*. *reuteri* (former*: Lb. reuteri*) [18,19], *Lpb*. *plantarum* subsp. *plantarum* (former*: Lb. plantarum*) [20,21], and *Lev. hammesii* (former *Lb. hammesii*) [22], in which different antifungal metabolites such as organic acids (lactic acid, acetic acid, phenyllactic acid) [23], fatty acid (monohydroxy C_18:1_) [22], or cyclic dipeptides [20] were detected. Fewer studies investigated antifungal activities of *Leuconostoc* strains and their application as non-conventional starter culture in sourdough fermentations [24,25,26]. However, considering the techno-functional activities of *Leuconostoc* strains, their use as even multifunctional starter cultures is promising to produce baked goods with less or no additives following a clean label strategy.

*Leuconostoc* strains are still described as non-conventional starter cultures [5]. However, they have increasingly gained attention due to their various functionalities, making them promising for application, e.g., in sugar reduction of soft buns as suggested by Sahin et al. [7]. So far, the focus has mainly been set on single functionalities, whereas this study examined LAB strains with multiple techno-functionalities that include mannitol production, antifungal activities, and EPS formation. With the aim to specifically suppress bakery-relevant fungal contaminants, the antifungal screening was carried out against fungal strains, isolated from an industrial bakery. This strategy led to the identification of promising *Leuconostoc citreum* strains with multiple techno-functional characteristics, i.e., mannitol production, EPS formation, and antifungal activities. Application of four selected *Lc. citreum* strains in lab-scale sourdough fermentations confirmed their assertiveness against the natural flour microbiota. Further, the monitoring of techno-functional metabolites production in sourdough allowed an evaluation for their suitability in sourdough fermentations and later application in bakery products.

## 2. Materials and Methods

### 2.1. Microbial Strains and Culture Conditions

LAB were isolated from two different wheat sourdoughs. All strains were routinely cultivated on de Man–Rogosa–Sharpe agar (MRS, Biokar, Beauvais Cedex, France) and incubated for 72 h at 30 °C (unless otherwise noted). For the isolation of LAB, 10 g of sourdough was mixed with diluent (1 g peptone and 8.5 g natrium chloride) 1:10 and plated on MRS agar, followed by incubation at 30 °C for 3 days. Randomly selected bacterial cultures (*n* = 5–15 colonies per dilution) were purified two times on MRS agar and identified by Matrix-Assisted Laser Desorption Ionization-Time of Flight Mass Spectrometry (MALDI-TOF MS, Bruker, Bremen, Germany), as described by Miescher Schwenninger et al. [27]. Extraction for MALDI-TOF MS was done by transferring single colonies to sterile double-distilled water followed by ethanol addition (300 µL). The supernatant obtained after centrifugation (13,000× *g*, 20 °C, 2 min) was removed and 20 µL of 70% acetic acid were added and the suspension was mixed. Then, the same amount of acetonitrile was added, mixed, and centrifugated as described above. Besides the LAB strains isolated in this study, a variety of different LAB strains that are part of the culture collection of the Food Biotechnology Research Group of ZHAW (Zurich University of Applied Sciences) and that were previously isolated from cereal-based products were used and identified by MALDI-TOF MS if necessary. All LAB used within this study are shown in Table 1.

### 2.2. Isolation and Identification of Fungal Contaminants from Industrial Bakery

In an industrial bakery, 58 fungal contaminants were isolated from air at ten different locations before and after the baking process using an air sampler equipped with either Dichloran-Bengalrot-Chloramphenicol (DRBC) agar or Dichloran Glycerin Chloramphenicol (DG18) agar. Further, fungi were selected from soft rolls produced without preservatives and stored until filamentous growth was visible. Fungal isolates were propagated on DRBC and DG18 agar at 25 °C until growth was visible, followed by 1–2 times purifying on the respective agar medium. In a first step, pure fungal cultures were classified according to their visual appearance on malt agar and Czapek Yeast Autolysate (CYA) agar, respectively, after growth at 25 °C for 7 days. In a second step, DNA of 1–2 representatives of each group (*n* = 16) was isolated and purified by using a Quick-DNA plant/seed miniprep Kit (D6020, Zymo Research, Irvine, CA, USA), followed by ITS sequencing, using standard primer ITS1 and ITS4 as described by White et al. [28]. Sequences were obtained by Microsynth (Balgach, Switzerland) and were evaluated using ITS database from NCBI blast: (https://blast.ncbi.nlm.nih.gov/Blast.cgi?PROGRAM=blastn&PAGE_TYPE=BlastSearch&LINK_LOC=blasthome; accessed on 27 June 2021).

### 2.3. Screening of LAB for Antifungal Activity against Moulds from A Bakery Environment

The antifungal screening was performed against *Aspergillus* (*A.*) *flavus* IB1 and *A. tritici* IB12, both isolated from soft rolls (this study). *Penicillium* (*P*.) *crustosum* FB005 was also isolated from soft rolls at the same industrial bakery as in this study [29], using an agar spot assay as described by Inglin et al. [30] with the following modifications. A wheat-based agar was used with wheat flour hydrolysate (WFH) that was prepared by mixing wheat flour (type 550, obtained from Meyerhans Mühlen AG, Weinfelden, CH) and water (1:5), followed by stirring at 90 rpm at 30 °C for 4 h and further cooling at 4 °C for 18 h prior to pH adjustment to 5.6 ± 0.2. For WFH agar, 15 g fructose, 15 g maltose, 15 g saccharose, 10 g yeast extract, and 18 g agar were mixed with 1 liter of wheat flour hydrolysate, followed by autoclaving (15 min, 121 °C). Then, 4 mL WFH agar were pipetted sterile into each cavity of a 6-well plate. For the antifungal screening, 10 mL of MRS broth were inoculated with one colony of a fully grown LAB culture and incubated at 30 °C for 17 h. Then, 2 μL of this culture were pipetted in the centre of the agar of each cavity and grown anaerobically at 30 °C for 72 h. After growth, 900 μL of soft malt agar (9% agar) inoculated with 4 × 10^6^ spores/mL of each one of the three moulds were poured onto each cavity. After further incubation at 25 °C for 3 to 5 days, zones of inhibition were evaluated by the following system: no inhibition (−); brightening of the medium (+/−); 0.1–2.4 mm halo (+); 2.5–4.9 mm halo (++); > 5 mm halo with minor fungal growth (++(+)); and complete inhibition of the fungi (+++). A first evaluation was done in a single run and LAB strains with antifungal activities were confirmed in triplicates. Further, the antifungal spectrum of nine *Leuconostoc* strains with high antifungal activities was determined against six moulds isolated from a bakery environment in this study and belonging to the genera *Cladosporium*, *Aspergillus*, and *Penicillium*, and a yeast (*Vishniacozyma*) using the agar spot assay as described above. *Leuconostoc* strains that inhibited fungal growth completely after the single run were confirmed in triplicates.

### 2.4. Screening of LAB for Mannitol Production

Mannitol production was screened in an MRS medium enriched with fructose (50 g/L), as described by Saha and Nakamura [31]. Therefore, strains were grown in MRS broth at 30 °C for 15 h. After incubation, cell counts were set to 1 × 10^6^ CFU/mL in MRS medium enriched with fructose (50 g/L) and incubated at 30 °C for 72 h. Samples of 10 mL (t_0_ and t_72_) were centrifuged (8000× *g*, 20 °C, 5 min) and sterile-filtered (0.2 µm) prior to HPLC analysis on an Agilent 1260 system using a Rezex RPM monosaccharide PB+2 column (300 × 7.8 mm, Phenomenex, Torrance, CA, USA) and a guard column (Carbo-Pb, Phenomenex, Torrance, CA, USA) coupled to an RI detector (1260 RID, G1362A, Agilent, Santa Clara, CA, USA; temperature set to 50 °C). Samples (5 µL) were eluted with double-distilled water at a flow rate of 0.6 mL/min and at a temperature of 85 °C.

### 2.5. Screening of LAB for EPS Production and Detection of Dextran Encoding Genes

A screening of LAB for EPS production was performed as described by Malang et al. [12] by estimating EPS production after growth on MRS agar enriched with 40 g/L sucrose for EPS production. Therefore, LAB strains were grown at 30 °C for 24 h in MRS broth. Then, 3 μL of the 24 h culture were pipetted onto the two agar media with different carbohydrate sources and incubated at 30 °C for 72 h. The produced slime was visually assessed, and the precipitation determined using 97% ethanol.

EPS positive strains of *Leuconostoc* sp. were further evaluated for the presence of genes encoding dextransucrase according to Coda et al. [14] using primer sets dsrM_F2 (5′-TTA GCA TCA TTA ACG AGA CCC-3′) and dsrM_R2 (5′-GCC AGT AAA GCC TTA TCA ACA G-3′), and dsrB_F2 (5′-GGT CAC TTC TGG CTT CAC TG-3′) and dsrB_R2 (5′-CCA TCA TTA CCC AAA TAG AAC CAC-3′). Briefly, 1 µL of each primer set was mixed with 12.5 µL of polymerase (KAPA Taq), 9.5 µL of double-distilled water, and 1 µL of template. The template was prepared by resuspending one colony in 100 µL of double-distilled water followed by heating up to 95 °C for 10 min. The PCR program was set as follows: 1 × (95 °C, 3 min); 35 × (95 °C, 30 s; 54 °C for dsrM_F2/dsrM_R2; 51 °C for dsrB_F2/dsrB_R2, 30 s; 72 °C, 30 s), 1 × (72 °C, 1 min). The amplified product was loaded on a 2% agarose gel having GelRed (Biotium) and run at 75 volts for 60 minutes, followed by gel analysis by an imaging system (Azure biosystems c300).

### 2.6. Lab-Scale Sourdough Fermentations with Selected Leuconostoc Strains

Lab-scale sourdough fermentations were prepared by mixing 200 g wheat flour (type 550, obtained from Meyerhans Mühlen AG, Weinfelden, CH) with sterile tap water (1:1), 1.5 g saccharose, and 1.5 g fructose (per 100 g flour) in a sterile beaker, followed by inoculation with 1 × 10^7^ CFU of pure cultures of *Lc. citreum* strains DCM49, DCM65, MA079, or MA113 per gram sourdough. Cultures were therefore washed twice with sterile tap water and sourdough fermentations were covered by aluminium foil. Cell counts before and after 24 h fermentation at 30 °C were determined by mixing 10 g sourdough 1:10 with dilution solution and further surface plating on MRS and plate count (PC) agar, followed by incubation at 30 °C for 3 days. At each sampling point and agar medium, 15 colonies were randomly selected and analysed by MALDI-TOF MS to ensure growth of the desired LAB species. The following MALDI-TOF MS scores were applied: 0−1.69 = not reliable identification, 1.70–1.99 = probable genus identification, and 2.00−2.29 = secure genus identification, probable species identification. Further, pH and total titratable acidity (TTA) were measured at t_0_ and t_24_. Acidification (pH) of the sourdough was measured by analysing the diluted sourdough (1:10), whereas TTA was measured by adding 5 mL of acetone to 10 g of sourdough and 95 mL of distilled water, followed by manual stirring before the measurement in a Titrino plus 848 (Methrom, Zofingen, Switzerland).

### 2.7. Determination of Antifungal Compounds in Sourdough

Antifungal compounds in sourdough were quantified as described by Brosnan et al. [32] with the following modifications. Sourdough samples (ca. 30 g) were centrifuged (8000× *g*, 20 °C, 5 min) and 5 mL of supernatant were mixed with 4 g of magnesium sulphate, 1 g of sodium chloride, and 10 mL of ethyl acetate with 1% formic acid. The solution was shaken for 1 minute followed by further centrifugation (3000× *g*, 20 °C, 10 min). The supernatant was added to a dSPE kit tube (Agilent, Santa Clara, CA, USA) and shaken for 1 min. Then, the tube was centrifuged (3000× *g*, 20 °C, 10 min), and the supernatant was mixed with 100 μL of dimethylsulfoxide and evaporated with nitrogen gas. The remaining sediment was mixed with 900 μL of a water/acetonitrile solution (90:10) and sterile-filtered. High-performance liquid chromatography (HPLC) was done on a HPLC system (Agilent 1260) that was equipped with a diodenarray detector (1260 DAD, G4212B) using a Gemini 5 µm C18 110 Å column (150 × 2 mm, Phenomenex, Torrance, CA, USA) and a guard column (C18, Phenomenex). Samples were eluted with double-distilled water with 1% formic acid (A) and acetonitrile with 1% formic acid (B) with the following gradient: 0 min, 95% A and 5% B; 5 min, 90% A and 10% B; 10 min, 85% A and 15% B; 20 min, 85% A and 15% B; 30 min, 80% A and 20% B; 31 min, 1% A and 99% B; 37 min, 1% A and 99% B; 42 min, 95% A and 5% B; 43 min, 95% A and 5% B; 45 min, 95% A and 5% B; and 50 min, 95% A and 5 % B at a flow rate of 0.2 mL/min. The temperature was not controlled. A volume of 10 µL was injected and detection was done at a wavelength of 210 nm. Hydroxyphenyllactic acid (HPLA), phenyllactic acid (PLA), benzoic acid, 4-Hydroxybenzoic acid, hydrocaffeic acid, caffeic acid, hydroxyphenylpropionic acid (HPPA), trans-ferulic acid, coumaric acid, and hydroferulic acid were used as antifungal reference compounds.

### 2.8. Determination of Fructose, Mannitol, and Acids in Sourdoughs

Contents of sugars (fructose and mannitol) and organic acids (lactic and acetic acids) in sourdough were analysed as described by Müller et al. [33]. Therefore, 1 g of sourdough was diluted with 5 mL of double-distilled water, followed by heating up to 80 °C for 3 h. After centrifugation (5000× *g*, 4 °C, 5 min), the supernatant was mixed 1:1 with 7% perchloric acid. This mixture was stored at 4 °C overnight. After further centrifugation (5000× *g*, 4 °C, 5 min), samples were sterile-filtered (0.2 μm) and analysed by HPLC. Mannitol and fructose were determined as described above. Lactic and acetic acids were analysed by using a ROA-Organic Acid H+ column (150 × 4.6 mm, Phenomenex, Torrance, CA, USA) and a guard column (Carbo-H, Phenomenex, Torrance, CA, USA) with 0.005 M sulphuric acid as eluent at a flow rate of 0.2 mL/min. The detection was done by DAD at 210 nm. The temperature of the column was set to 25 °C and a volume of 5 µL was injected. 

### 2.9. Determination of EPS in Sourdoughs

Microbial and flour EPS in sourdough were analysed as described by Galli et al. [34] with the following modifications. A defined sourdough aliquot was centrifuged (11,000× *g*, 4 °C, 10 min), the supernatant was mixed with a double volume of cold ethanol and stored at 4 °C overnight. Then, samples were centrifugated (2500× *g*, 5 °C, 20 min) and the precipitated EPS were dissolved in double-distilled water and again precipitated with cold ethanol. This step was repeated. Finally, precipitated EPS were dried in a drying oven at 105 °C for 3 h and the quantity was determined gravimetrically by an analytical scale.

### 2.10. Statistical Analysis

All experiments were performed in triplicates, unless otherwise stated. Dataset was analysed for normal distribution using Shapiro–Wilk test and/or was visually evaluated by Q–Q plot. If data were normally distributed, a one-way ANOVA and Tukey HSD Test was applied as post hoc test. ANOVA was performed in RStudio (version 1.4.1106) at a significance level of *p* ≤ 0.05. For not normally distributed data, a nonparametric Kruskal–Wallis test was applied, following by Wilcoxon test as post hoc test (unpaired).

## 3. Results

### 3.1. Isolated LAB Strains from Cereal-Based Products

In total, 132 strains were isolated from two wheat sourdoughs using MRS agar. MALDI-TOF MS identification revealed *Lc. citreum* (54×), *Lc. mesenteroides* (1×), *Lc. pseudomesenteroides* (1×), *Lc. palmae* (2×), *Lac. paracasei* subsp. *paracasei* (43×), *L*. *lactis* (19×), *Llb*. *curvatus* (8×), *F. fructivorans* (2×), *Lo. coryniformis* subsp*. coryniformis* (1×), and *W. confusa* (1×).

### 3.2. Analysis of the Fungal Microbiota in an Industrial Bakery

In total, 58 fungal contaminants were isolated in an industrial bakery, either from air or from soft rolls produced without preservatives and stored at room temperature for 6 days. The isolates were further compiled to 16 groups based on their appearance on malt agar and CYA agar and, finally, 1−2 examples of each group were identified by sequence analyses of the ITS region. As shown in Table 2, a dominance of *Aspergillus* sp. (4 groups with 17 isolates), *Penicillium* sp. (5 groups with 24 isolates), and *Cladosporium* sp. (2 groups with 8 isolates) was determined within the mould contaminants. *Aspergillus* sp. was isolated before and after the baking process and, in contrast, *Cladosporium* sp., *Penicillium* sp., *Aureobasidium* sp., and *Byssochlamys* sp. were only isolated after the baking process. Yeast contaminants comprising *Vishniacozyma* sp., *Rhodotorula* sp., and *Hannaella* sp. were only isolated from facility air.

### 3.3. Antifungal Activities of LAB Determined on A Cereal-Based Medium

In a first step, a high-throughput screening of 99 LAB strains against *P*. *crustosum* FB005, *A. flavus* IB1, and *A. tritici* IB12 (both this study) was done on a cereal-based (WFH) medium (Table 3). Sensitivity of the three moulds was determined as follows: (I) *P*. *crustosum* FB005 was only very weakly suppressed determined by no clear halo formation; (II) *A. flavus* IB1 was generally moderately suppressed shown by halos of up to 2.4 mm by strains of *Lev. brevis*, *Li. fermentum*, *Le. parabuchneri*, *Lc. citreum*, *Lc. mesenteroides*, *Lc. palmae*, and *Lc. pseudomesenteroides;* and (III) *A. tritici* IB12 was strongly suppressed by 53 *Leuconostoc* sp. strains shown by halos of a radius of >5 mm. In a second step, based on the high-throughput screening, *Leuconostoc* strains with the highest antifungal activities (*n* = 9) were selected and screened on WFH medium against *Cladosporium* sp. strains B1 and F40, *Penicillium* sp. strains F27 and F43, and *Aspergillus* sp. F18 as representatives of contaminating moulds and *Vishniacozyma* sp. F9 as an example for a contaminating yeast. All tested strains of *Leuconostoc* sp. were able to inhibit the two strains of *Cladosporium* sp. (B1 and F40) and *Vishniacozyma* sp. F9 (Table 4). *Lc. citreum* DCM49 was the only strain that fully inhibited *Aspergillus* sp. F18 but only weakly inhibited both *Penicillium* strains (F27 and F43). In contrast, *Lc. citreum* DCM65 was the only strain that fully suppressed both *Penicillium* sp. strains (F27 and F43), but only weakly suppressed *Aspergillus* sp. F18. Further, *Leuconostoc* strains MA113, DCM83, and DCM27 were able to fully suppress *Penicillium* sp. strain F27 but to a lesser extent suppressed *Penicillium* sp. F43 and *Aspergillus* sp. F18.

### 3.4. Exopolysaccharides Production by LAB and Detection of Dextransucrase Genes

In a high-throughput screening on MRS-enriched sucrose medium strains of *Lc. citreum* (56), *Lc. mesenteroides* (3), *Lc. palmae* (2), and *W. confusa* (1) showed slimy colonies that precipitated immediately with ethanol addition indicating exopolysaccharides (EPS) production. No slime was detected in strains of *Lev. brevis* (73), *Lac. paracasei* subsp. *paracasei* (58), *P. pentosaceus* (43), *Lpb. plantarum* subsp. *plantarum* (33), *L. lactis* (19), *Llb. curvatus* (11), *Lo. coryniformis* subsp. *coryniformis* (6), *Le. parabuchneri* (4), *C. paralimentarius* (2), and *C. kimchii* (3). All *Leuconostoc* sp. strains were further analysed for presence or absence of *dsrM* and *dsrB* that are known genes encoding dextransucrase. At least one of these genes was present in all tested *Leuconostoc* strains (Table 5), *dsrB* was present in all tested *Lc. citreum* strains, and *dsrM* was present in 28 of the 56 tested strains of *Lc. citreum*.

### 3.5. Mannitol Production of Lactic acid Bacteria

In total, 69 out of 137 strains belonging to the species Lev. brevis, F. sanfranciscensis, Li. fermentum, Le. parabuchneri, Fu. rossiae, Lc. citreum, Lc. palmae, Lc. mesenteroides, and Lc. pseudomesenteroides showed mannitol production > 20 g/L in MRS supplemented with 50 g/L fructose (Table 6). All 54 strains of Leuconostoc citreum were mannitol producers, including 43 strains that yielded over 30 g/L mannitol in MRS medium.

### 3.6. Production of Techno-Functional Metabolites in Lab-Scale Sourdoughs Inoculated with Lc. citreum Strains DCM49, DCM65, MA079, or MA113

Antifungal-, mannitol-, and EPS-producing *Lc. citreum* strains DCM49, DCM65, MA079, and MA113 were applied as pure cultures in 400 g lab-scale sourdough fermentations in beakers. As a negative control, a sourdough without inoculation (spontaneously fermented) was prepared. All sourdoughs with culture addition developed up to 8.9−9.2 log CFU of presumptive LAB/g within 24 h, whereas in the spontaneously fermented sourdough, presumptive LAB developed only up to 7.5 log CFU/g (Table 7). Uniform colony morphology was detected visually on MRS and PCA plates after incubation at 30 °C for 72 h in sourdoughs started with *Lc. citreum* strains DCM49, DCM65, MA079, and MA113. In total, 15 isolates collected on MRS and PCA from samples before (t_0_) and after (t_24_) fermentation of inoculated sourdoughs were confirmed as *Lc. citreum* with MALDI-TOF MS scores between 1.72 and 2.21 at a level of 7 log CFU/g and 9 log CFU/g, respectively. In spontaneously fermented sourdough, six isolates were not identified (MALDI-TOF MS scores < 1.70). The remaining isolates were identified as non-LAB species, with most frequent occurrence of *Cronobacter* (*C*.) *sakazakii* (6 out of 15, with MALDI-TOF MS scores between 1.73 and 2.09), one *Enterobacter* (*E*.) *cloacae*, and one *E. asburiae* isolated on PCA (MALDI-TOF MS scores of 1.81 and 2.03, respectively). In addition, one LAB strain identified as *Llb. curvatus* with a MALDI-TOF MS score of 1.72 was isolated from spontaneous fermented sourdough. Before fermentations (t_0_; with and without inoculation), pH values between 6.2 and 6.4 were measured that dropped to 4.0–4.1 at 30 °C after 24 h in sourdoughs with inoculation, contrary to 5.6 in the spontaneously fermented sourdough. TTA in sourdoughs before fermentation (t_0_) was 2.1–2.6 (with and without inoculation) and increased after 24 h of 30 °C fermentation to 11.6–12.4 (depending on the inoculated *Lc. citreum* strain), whereas TTA of the spontaneously fermented sourdough only increased to 5.5.

Before the fermentation (t_0_), lactic acid, acetic acid, and mannitol were below the detection limit in all sourdoughs. After 24 h of fermentation, lactic and acetic acids were determined at concentrations of up to 3.3 mg/g and 1.7 mg/g, respectively, in sourdoughs inoculated with *Lc. citreum* strains, in contrast to 0.4 mg/g and 0.2 mg/g, respectively, in the spontaneously fermented sourdough. The fructose content decreased in all sourdoughs inoculated with *Lc. citreum* strains but did not change in the spontaneously fermented sourdough (9.2 mg/g sourdough at t_24_). In *Lc. citreum* inoculated sourdoughs, mannitol increased to 7.8–8.6 mg/g, whereas no mannitol was determined in spontaneously fermented sourdough at t_24_. After 24 h of fermentation, the antifungal compounds HPLA and PLA were found in sourdough fermentations inoculated with *Lc. citreum* strains DCM49, DCM65, MA079, or MA113 at levels of 0.6–1.3 μg/g and 1.3–2.1 μg/g, respectively, whereas lower amounts of HPLA (0.2 μg/g) and PLA (0.7 μg/g) were determined in spontaneously fermented sourdough. At t_0_, EPS were determined at a concentration of 0.07–0.16 g/100 g in all sourdoughs (independent of the inoculation) that increased to 0.25–0.62 g/100 g (*Lc. citreum* inoculated) and 0.77 g/100 g (spontaneous fermentation).

## 4. Discussion

This study assessed the use of *Lc. citreum* as non-conventional LAB starter cultures with techno-functional characteristics and their suitability in sourdough fermentations. By applying a multifunctional starter culture that combines multiple techno-functional characteristics, the produced sourdough has high potential as a natural functional ingredient for baked goods [7]. This approach goes along with a clean label strategy aiming at reducing food additives (E-numbers) and following consumer’s demand for more naturally and gently produced food.

Fungal contaminations of baked goods are a major issue bakeries have to face, often leading to financial losses [35]. If mycotoxigenic moulds are involved, it even endangers human health [36]. Aerocontamination by spores from bakery-relevant moulds such as *Penicillium*, *Eurotium*, *Aspergillus*, and *Cladosporium* have been described as a reason for the spoilage of bread [29,37]. This usually occurs after the baking process during cooling, slicing, or storage of the soft bread [35]. Similar, typical bakery-relevant moulds including *Aspergillus*, *Penicillium*, and *Cladosporium* were isolated in this study from the facility’s air, whereas most isolates belonged to the genera *Penicillium* (24 isolates), *Aspergillus* (17 isolates), and *Cladosporium* (8 isolates). Furthermore, *Byssochlamys*, *Aureobasidium*, and yeasts (*Hannaella*, *Vishniacozyma*, and *Rhodotorula*) were found in the facility’s air. On soft breads, moulds of the genera *Penicillium* sp. and *Cladosporium* sp. were isolated. Since in this study *Penicillium* sp. and *Cladosporium* sp. were only isolated at locations after the baking process, it is assumed that the soft rolls were contaminated after the heating process, e.g., by aerocontamination, as mentioned by Santos et al. [38]. So far, there are different methods to overcome fungal contaminations in the bakery context such as the use of ethanol and modified atmosphere packaging [15]. A natural approach is the application of LAB strains with antifungal activities with the aim to replace or reduce food additives in future [39]. In order to find suitable antifungal starter cultures specifically acting against fungal spores within the baking environment, the dominate fungal flora of a bakery should be monitored as an initial step. A similar fungal monitoring was previously described by Freimüller Leischtfeld and Miescher Schwenninger [29], where it was shown that the main fungal contaminants belonged to *Cladosporium* sp., *Aspergillus* sp., and *Penicillium* sp. So far, strains of *Leuconostoc* sp. were less considered as antifungal cultures in sourdough fermentations compared to the well-studied species of the previous genera *Lactobacillus* reclassified to *Lb. amylovorus*, *Li. reuteri* (former: *Lb. reuteri*), and *Lpb. plantarum* subsp. *plantarum* (former: *Lb. plantarum*) [16,20,40]. This study showed that strains of the genus *Leuconostoc* (mainly *Lc. citreum*) revealed strong inhibition of bakery-relevant moulds such as *Aspergillus*, *Cladosporium*, and *Penicillium* on a cereal-based medium and are thus promising strains for application in biocontrol concepts of bakery products.

The metabolisms of heterofermentative LAB such as *Leuconostoc* sp. are well studied, and it is known that fructose is reduced followed by the formation of mannitol by a mannitol-dehydrogenase that is connected to the formation of acetate [41]. It is therefore a balancing act between mannitol production and the formation of acetic acid, of which the latter can impact sensory [33]. The present study demonstrates that mannitol produced by 62 tested strains of *Leuconostoc* sp. varied between 20 g/L and up to over 30 g/L, and almost 80% of the tested *Lc. citreum* strains produced over 30 g/L. Furthermore, strains of *Leuconostoc* sp. were shown to produce EPS that positively influence the bread texture, as reviewed by Zannini et al. [42]. In this study, EPS formation was detected in mannitol-producing *Lc. citreum* strains showing high potential for multiple functionalities. Further, all *Leuconostoc* strains harbored at least one gene-encoding dextransucrase of which all *Lc. citreum* strains showed presence of *dsrB*. Coda et al. [14] investigated five genes encoding dextransucrase and showed that the presence of *dsrB* was mainly responsible for the formation of dextran and that *dsrB* was the only gene that was significantly upregulated during sourdough fermentation started with *Lc. citreum* FDR241. It is therefore assumed that, among others, dextran is produced by the *Lc. citreum* strains investigated in this study.

Four selected *Lc. citreum* strains, DCM49, DCM65, MA079, and MA113, with high antifungal activities, mannitol production, and EPS formation, were finally applied in lab-scale sourdough fermentations. The metabolic activity of the strains in sourdough, and thus their suitability as potential techno-functional starter cultures, was evaluated based on LAB growth, acidification, fructose reduction, mannitol production, EPS formation, and contents of antifungal compounds and was compared with a spontaneously fermented sourdough. The development of LAB up to 9.2 log CFU/g during 24 h at 30 °C and the confirmation of the presence of *Lc. citreum* by MALDI-TOF MS up to log 9 CFU/g, pH reduction from 6.4–6.2 to 4.0–4.1, TTA increase from 2.2–2.6 to 11.6–12.4, and production of lactic (3.0–3.3 mg/g) and acetic (1.5–1.7 mg/g) acids indicated the assertiveness of *Lc. citreum* strains DCM49, DCM65, MA079, and MA113 against the naturally occurring sourdough microflora. Acetic acid was described by Quattrini et al. [43] as the most relevant compound produced by LAB against fungal contaminants. Axel et al. [40] used the following minimum inhibitory concentrations (MIC) references at pH 5: > 45 mg/mL for lactic acid and 1.2–7.2 mg/mL for acetic acid as. In this study, lactic acid (3.0–3.3 mg/g) was clearly below this MIC reference, whereas acetic acid concentrations (1.5–1.7 mg/g) were within the MIC range. Besides lactic and acetic acids, minor amounts of further carboxylic acids were detected with up to 2.1 µg/g PLA and 1.3 µg/g HPLA. Their effect in the end product, e.g., in a sourdough bread, was described as too low by Axel et al. [40], using MIC references of 2500–10,000 µg/g for PLA and 5000 µg/g for HPLA. They hypothesized synergistic effects as being responsible for mould inhibition under acidic conditions [40]. Thus, there is still more research needed to better understand antifungal activities of LAB. Further, in lab-scale sourdough fermentations inoculated with the selected *Lc. citreum* strains, fructose was reduced and mannitol was produced in a range between 7.8 and 8.6 mg/g. In comparison, spontaneously fermented sourdough showed weak LAB development of only up to 7.5 log CFU/g, pH decrease from 6.3 to 5.6, TTA increase from 2.1 to 5.6, and only small production of lactic (0.4 mg/g) and acetic (0.2 mg/g) acids after 24 h fermentation. The conditions of the spontaneously fermented sourdough might have triggered the growth of the opportunistic foodborne pathogen *C. sakazakii* that was also isolated by Lou et al. [44] from cereal-based products. Since, in this study, no added fructose was metabolized during the spontaneous sourdough fermentation, it is assumed that the flora of the spontaneously fermented sourdough was not able to reduce fructose. Small amounts of EPS (0.07–0.16 g/100 g sourdough) were determined before the fermentation (t_0_) and independent of *Lc. citreum* inoculation or no inoculation, which could be due to naturally occurring polysaccharides in flour as described by Garófalo et al. [45]. Amounts of EPS slightly increased in fermented sourdoughs with and without inoculum up to 0.62 g/100 g and 0.77 g/100 g, respectively. The EPS produced in the spontaneously fermented sourdough may have been formed by *C. sakazakii* and or *Enterobacter* sp. *C. sakazakii* [46] and *Enterobacter* sp. [47] are known to produce EPS.

## 5. Conclusions

In this study, techno-functional characteristics, i.e., antifungal activities, mannitol production, and EPS formation of *Lc. citreum* strains DCM49, DCM65, MA079, and MA113, were observed in screening assays. It was confirmed in lab-scale sourdough fermentations that all four *Lc. citreum* strains produced antifungal compounds (e.g., acetic acid, PLA, and HPLA), mannitol, and EPS. In summary, this study showed that *Leuconostoc* and specifically *Lc. citreum* have high potential as multiple techno-functional starter cultures in sourdough fermentations. Multiple functional sourdoughs as described in this study can be of interest since this may have beneficial applications in clean label strategies for bakery products.

## Figures and Tables

**Table 1:** Overview of LAB strains used in this study.

Genus and Species ^1^	Former Genus (Incl. Species)	Total No. of Isolates	No. of Isolates Used in Screenings			Isolation Source	References
			Antifungal	EPS	Mannitol		
*Lev. brevis*	*Lb. brevis*	73	4	73	3	Wheat sourdough/Rye sourdough	ZHAW collection ^2^
*Lacticaseibacillus* (*Lac.*) *paracasei* subsp. *paracasei*	*Lb. paracasei*	58	2	58	3	Wheat sourdough/Rye sourdough	This study/ ZHAW collection
*Li. fermentum*	*Lb. fermentum*	1	1	- ^3^	1	Baker’s yeast	ZHAW collection
*Lpb. plantarum* subsp. *plantarum*	*Lb. plantarum*	33	10	33	12	Malt/baker’s yeast	ZHAW collection
*Lactococcus* (*L.*) *lactis*		19	1	19	19	Wheat soudough	This study
*Latilactobacillus* (*Llb.*) *curvatus*	*Lb. curvatus*	11	-	11	8	Wheat sourdough/baker’s yeast	This study/ ZHAW collection
*Loigolactobacillus* (*Lo*.) *coryniformis* subsp. *coryniformis*	*Lb. coryniformis*	6	-	6	4	Wheat sourdough	This study/ ZHAW collection
*Lentilactobacillus* (*Le.*) *parabuchneri*	*Lb. parabuchneri*	4	1	4	1	Rye sourdough	ZHAW collection
*Lb. delbrueckii* subsp. *delbrueckii*	*Lb. delbrueckii*	1	-	-	1	Brewer’s spent grain	ZHAW collection
*Compani-lactobacillus* (*C.*) *paralimentarius*	*Lb. paralimentarius*	2	-	2	-	Rye sourdough	ZHAW collection
*C. kimchii*	*Lb. kimchii*	3	-	3	1	Rye sourdough	ZHAW collection
*F. fructivorans*	*Lb. fructivorans*	2	1	-	2	Wheat sourdough	This study
*F. sanfranciscensis*	*Lb. sanfranciscenis*	2	1	-	2	Rye sourdough	ZHAW collection
*Furfurilactobacillus* (*Fu.)* *rossiae*	*Lb. rossiae*	1	-	-	1	Rye sourdough	ZHAW collection
*Pediococcus (P.) pentosaceus*		43	-	43	2	Wheat sourdough	ZHAW collection
*Lc.* *lactis*		14	-	-	14	Brewer’s spent grain	ZHAW collection
*Lc. citreum*		68	68	56	54	Wheat sourdough/malt	This study/ ZHAW collection
*Lc. mesenteroides*		5	3	3	5	Wheat sourdough/baker’s yeast	This study/ ZHAW collection
*Lc. pseudo-mesenteroides*		3	3	-	1	Wheat sourdough/malt	This study/ ZHAW collection
*Lc. palmae*		2	2	2	2	Wheat sourdough	This study
*W. confusa*		2	2	1	1	Wheat sourdough/malt	This study/ ZHAW collection

^1^ Classification according to Zheng et al. [2]. ² Strains obtained from ZHAW culture collection (Food Biotechnology Research Group). ³ Not applied in the screening (-).

**Table 2:** Representative strains of 16 groups of 58 fungal contaminants isolated from ten different locations from facility air and soft rolls (produced without preservatives) and identified by sequence analyses of the ITS region.

Macroscopic Groups	No. of Isolates per Group	Genus/Species	Identified Strain	Isolation Location
		**Isolated before baking**		
1	2	*Vishniacozyma victoriae*	F9	Facility air
2	5	*Aspergillus* sp. (*A. austwickii/A. aflatoxiformans*) ^2^	F17	Facility air
3	3	*Aspergillus* sp. (*A. tennesseensis/A. jensenii*) ^2^	F18	Facility air
		**Isolated after baking**		
4	2	*Aspergillus welwitschiae*	F32	Facility air
5	7	*Aspergillus* sp. (*A. austwickii/A. aflatoxiformans*) ^2^	F55	Facility air
6	4	*Penicillium* sp. (*P. fuscoglaucum/P. palitans/P. commune*) ^2^	F26	Facility air
7	3	*Penicillium chrysogenum*	F27	Facility air
8 ^1^	11	*Penicillium rubens* *Penicillium sp.(P. fuscoglaucum/P. palitans)* ^2^	F43 B3	Facility air Soft rolls
9	4	*Penicillium* sp. (*P. fuscoglaucum/P. palitans/P. commune*) ^2^	F66	Facility air
10	2	*Penicillium rubens*	B4	Soft rolls
11	1	*Cladosporium antarcticum*	F61	Facility air
12 ^1^	7	*Cladosporium* sp. (*C. subcinereum/C. antarcticum*) ^2^ *Cladosporium domesticum*	B1 F40	Soft rolls Facility air
13	1	*Aureobasidium melanogenum*	F47	Facility air
14	3	*Byssochlamys spectabilis*	F57	Facility air
15	1	*Hannaella oryzae*	F33	Facility air
16	2	*Rhodotorula mucilaginosa*	F20	Facility air

^1^ Two isolates per group. ² Species in brackets could not be distinguished by the method applied; appropriate identification is only possible on genus level.

**Table 3:** Antifungal activities of 99 LAB strains determined on WFH medium against *P*. *crustosum* FB005, *A. flavus* IB1, and *A. tritici* IB12 all isolated from soft rolls. Numbers in rows represent numbers of antifungal strains; zones of inhibition (halo measured from edge of colony to edge of zone of inhibition): +++ = complete inhibition of fungi; ++(+) = > 5 mm with minor fungi growth; ++ = 2.5–4.9 mm; + = 0.1–2.4 mm; +/- = no halo but a brightening of the medium; and - = no inhibition. If inhibition was detected in the single run, screening was performed in triplicates.

		Mould Contaminants					
		*P. crustosum* FB005		*A. flavus* IB1		*A. tritici* IB12	
Genus and Species	Total Strains	Inhibition	No. of Strains	Inhibition	No. of Strains	Inhibition	No. of Strains
*Lev. brevis*	4	- ^1^	4	− +	3 1	+++	4
*Li. fermentum*	1	+/−	1	+	1	++(+)	1
*F. fructivorans*	1	-	1	-	1	-	1
*Le. parabuchneri*	1	+/−	1	+	1	+++	1
*Lac. paracasei* subsp. *paracasei*	2	-	2	-	2	+	2
*Lpb. plantarum* subsp. *plantarum*	10	-	10	-	10	+/− +	1 9
*F. sanfranciscensis*	1	-	1	-	1	-	1
*L. lactis*	1	-	1	-	1	+/−	1
*Lc. citreum*	68	- +/-	62 6	- +	64 4	+ ++ ++(+) +++	3 17 8 40
*Lc. mesenteroides*	3	-	3	- +	2 1	+ ++ ++(+)	1 1 1
*Lc. palmae*	2	− +/−	1 1	− +	1 1	+++	2
*Lc. pseudo-mesenteroides*	3	-	3	− +	2 1	+/− +++	1 2
*W. confusa*	2	-	2	-	2	++(+) +++	1 1

^1^ single run if no inhibition (-) was detected.

**Table 4:** Inhibition spectrum of nine *Leuconostoc* strains determined on WHF medium against six fungal contaminants of a bakery environment with *Cladosporium* sp., *Penicillium* sp., and *Aspergillus* sp. as representative moulds and *Vishniacozyma* sp. as yeast example. *Cladosporium* sp. B1 was isolated from soft rolls, whereas the other fungal contaminants were isolated from facility air. Zones of inhibition (halo measured from edge of colony to edge of zone of inhibition): +++ = complete inhibition of fungi; ++(+) = > 5 mm with minor fungi growth; ++ = 2.5–4.9 mm; + 0.1–2.4 mm; +/- = no halo but a brightening of the medium; and - = no inhibition. Experiments were performed in triplicates if the mould was fully inhibited in the first run.

	Isolated Fungi from Bakery Environment					
Genus/Species	*Cladosporium* sp. B1 ^1^	*Cladosporium domesticum* F40	*Vishniacozyma victoriae* F9	*Penicillium chrysogenum* F27	*Penicillium rubens* F43	*Aspergillus* sp. F18 ^2^
*Lc. citreum* DCM49	+++	+++	+++	+ ^3^	+/− ^3^	+++
*Lc. citreum* DCM65	+++	+++	+++	+++	+++	+ ^3^
*Lc. citreum* MA113	+++	+++	+++	+++	++	++ ^3^
*Lc. citreum* MA079	+++	+++	+++	++(+) ^3^	++	++(+)
*Lc. citreum* DCM63	+++	+++	+++	++(+) ^3^	+/− ^3^	++ ^3^
*Lc. citreum* DCM74	+++	+++	+++	+ ^3^	++	++(+)
*Lc. citreum* DCM83	+++	+++	+++	+++	+/− ^3^	+ ^3^
*Lc. mesenteroides* DCM27	+++	+++	+++	+++	++	+ ^3^
*Lc. palmae* DCM85	+++	+++	+++	+ ^3^	++ ^3^	+ ^3^

^1^ Identified as *C. subcinereum*/*C. antarcticum*, appropriate identification is only possible on genus level by the method applied. ^2^ Identified as *A. tennesseensis/A. jensenii*, appropriate identification is only possible on genus level by the method applied. ^3^ Single run if no inhibition (-) was detected.

**Table 5:** EPS production of 59 *Leuconostoc* sp. strains on MRS supplemented with sucrose (40 g/L) and presence of dextransucrase genes *dsrM* (fragment size: 221 bp) and *dsrB* (fragment size: 192).

LAB Strains	Slimy Colony	Precipitation with ETOH ^1^	Dextransucrase-Encoding Genes			
			*dsrM*		*dsrB*	
			Present	Absent	Present	Absent
*Lc. citreum* (56)	+	+	28	28	56	0
*Lc. mesenteroides* (1)	+	+	0	1	1	0
*Lc. palmae* (2)	+	+	0	2	2	0

^1^ Slime precipitation confirmed EPS (+), and genes encoding for dextransucrase were amplified by using primer sets dsrM_F2/dsrM_R2 and dsrB_F2/dsrB_R2.

**Table 6:** Mannitol production of LAB in MRS medium supplemented with fructose (50 g/L) incubated at 30 °C for 72 h.

		Mannitol		
Genus/Species	No. of Strains Tested	0–20 g/L	20–30 g/L	>30 g/L
*L. lactis*	19	19 ^1^		
*Lpb. plantarum* subsp. *plantarum*	12	12		
*Llb. curvatus*	8	8		
*Lo. coryniformis* subsp. *coryniformis*	4	4		
*Lac. paracasei* subsp. *paracasei*	3	3		
*Lev. brevis*	3	1		2
*F. fructivorans*	2	2		
*F. sanfranciscensis*	2		1	1
*P. pentosaceus*	2	2		
*Lb. delbrueckii* subsp*. delbrueckii*	1	1		
*Li. fermentum*	1			1
*C. kimchii*	1	1		
*Le. parabuchneri*	1		1	
*Fu. rossiae*	1		1	
*W. confusa*	1	1		
*Lc. lactis*	14	14		
*Lc. citreum*	54		11	43
*Lc. palmae*	2		1	1
*Lc. mesenteroides*	5		1	4
*Lc. pseudomesenteroides*	1			1

^1^ Numbers in columns three to five show the number of mannitol-producing strains.

**Table 7:** Cell counts, pH, TTA, antifungal compounds, acids, fructose, mannitol, and EPS in lab-Scheme 400 g, 30 °C, 24 h) inoculated with four *Lc. citreum* strains and a spontaneously fermented sourdough without inoculation at t_0_ (before) and t_24_ (after fermentation for 24 h at 30 °C). Given values are average values ± standard deviation.

		*Lc. citreum* DCM49	*Lc. citreum* DCM65	*Lc. citreum* MA079	*Lc. citreum* MA113	No Inoculum
**LAB** **(log CFU/g)**	t_0_	7.2 ± 0.0 ^a^	7.1 ± 0.3 ^a^	7.2 ± 0.1 ^a^	7.2 ± 0.2 ^a^	<5 ^b^
	t_24_	9.2 ± 0.1 ^a^	9.1 ± 0.1 ^ab^	9.0 ± 0.1 ^ab^	8.9 ± 0.1 ^b^	7.5 ± 0.1 ^c^
**pH**	t_0_	6.4 ± 0.0 ^a^	6.4 ± 0.1 ^a^	6.3 ± 0.0 ^a^	6.2 ± 0.0 ^a^	6.3 ± 0.0 ^a^
	t_24_	4.0 ± 0.0 ^a^	4.0 ± 0.0 ^a^	4.0 ± 0.0 ^a^	4.1 ± 0.0 ^a^	5.6 ± 0.2 ^b^
**TTA**	t_0_	2.2 ± 0.1 ^b^	2.3 ± 0.1 ^ab^	2.4 ± 0.1 ^ab^	2.6 ± 0.2 ^a^	2.1 ± 0.1 ^b^
	t_24_	12.4 ± 0.1 ^b^	12.1 ± 0.1 ^ab^	11.6 ± 0.3 ^a^	11.8 ± 0.3 ^ab^	5.5 ± 0.2 ^c^
**Antifungal compounds (** **μg/g sourdough)**						
**HPLA**	t_0_	n.d.	0.0 ± 0.1 ^a^	n.d.	0.2 ± 0.2 ^a^	n.d.
	t_24_	1.1 ± 0.2 ^ab^	0.6 ± 0.5 ^ab^	1.3 ± 0.1 ^a^	1.2 ± 0.6 ^a^	0.2 ± 0.3 ^b^
**PLA**	t_0_	n.d.	n.d.	n.d.	n.d.	n.d.
	t_24_	1.6 ± 0.4 ^ab^	1.8 ± 0.5 ^a^	2.1 ± 0.4 ^a^	1.3 ± 0.0 ^ab^	0.7 ± 0.7 ^b^
**Acids (mg/g sourdough)**						
	t_0_	n.d.	n.d.	n.d.	n.d.	n.d.
**Lactic acid**	t_24_	3.0 ± 0.0 ^a^	3.1 ±0.0 ^a^	3.1 ± 0.3 ^a^	3.3 ± 0.4 ^a^	0.4 ± 0.1 ^b^
	t_0_	n.d.	n.d.	n.d.	n.d.	n.d.
**Acetic acid**	t_24_	1.6 ± 0.0 ^a^	1.5 ± 0.0 ^a^	1.7 ± 0.1 ^a^	1.6 ± 0.1 ^a^	0.2 ± 0.1 ^b^
**Carbohydrates (mg/g sourdough)**						
**Fructose**	t_0_	10.0 ± 0.9 ^a^	9.7 ± 1.9 ^a^	10.5 ± 1.7 ^a^	8.2 ± 1.7 ^a^	9.3 ± 1.0 ^a^
	t_24_	n.d.	2.03 ± 0.1 ^a^	n.d.	2.4 ± 0.3 ^a^	9.2 ± 0.5 ^b^
	t_0_	n.d.	n.d.	n.d.	n.d.	n.d.
**Mannitol**	t_24_	7.8 ± 0.5 ^a^	8.3 ± 0.2 ^a^	7.9 ± 0.8 ^a^	8.6 ± 1.0 ^a^	n.d.
**EPS formation (g/100 g sourdough**						
	t_0_	0.09 ± 0.05 ^ab^	0.13 ± 0.05 ^ab^	0.16 ± 0.01 ^a^	0.07 ± 0.01 ^b^	0.11 ± 0.01 ^ab^
	t_24_	0.36 ± 0.04 ^a^	0.45 ± 0.46 ^a^	0.25 ± 0.06 ^a^	0.62 ± 0.37 ^a^	0.77 ± 0.19 ^a^

n.d.: not detected molecules. a–c ANOVA or Kruskal–Wallis test followed by post hoc test (Tukey HSD Test/Wilcoxon) were carried out for each measurement and point in time (t0 and t24). Values in one row following the same letter are not significantly different.

## References

[1] Leroy F., De Vuyst L. (2004). Lactic acid bacteria as functional starter cultures for the food fermentation industry. Trends Food Sci. Technol..

[2] Zheng J., Wittouck S., Salvetti E., Franz C.M.A.P., Harris H.M.B., Mattarelli P., O’Toole P.W., Pot B., Vandamme P., Walter J. (2020). A taxonomic note on the genus *Lactobacillus*: Description of 23 novel genera, emended description of the genus *Lactobacillus* Beijerinck 1901, and union of *Lactobacillaceae* and *Leuconostocaceae*. Int. J. Syst. Evol. Microbiol..

[3] Arora K., Ameur H., Polo A., Di Cagno R., Rizzello C.G., Gobbetti M. (2021). Thirty years of knowledge on sourdough fermentation: A systematic review. Trends Food Sci. Technol..

[4] De Vuyst L., Van Kerrebroeck S., Harth H., Huys G., Daniel H.-M., Weckx S. (2014). Microbial ecology of sourdough fermentations: Diverse or uniform?. Food Microbiol..

[5] Montemurro M., Celano G., De Angelis M., Gobbetti M., Rizzello C.G., Pontonio E. (2020). Selection of non-Lactobacillus strains to be used as starters for sourdough fermentation. Food Microbiol..

[6] Wisselink H.W., Weusthuis R.A., Eggink G., Hugenholtz J., Grobben G.J. (2002). Mannitol production by lactic acid bacteria: A review. Int. Dairy J..

[7] Sahin A.W., Rice T., Zannini E., Axel C., Coffey A., Lynch K.M., Arendt E.K. (2019). *Leuconostoc citreum* TR116: In-situ production of mannitol in sourdough and its application to reduce sugar in burger buns. Int. J. Food Microbiol..

[8] Chen X.Y., Levy C., Gänzle M.G. (2016). Structure-function relationships of bacterial and enzymatically produced reuterans and dextran in sourdough bread baking application. Int. J. Food Microbiol..

[9] Bounaix M.-S., Gabriel V., Morel S., Robert H., Rabier P., Remaud-Siméon M., Gabriel B., Fontagné-Faucher C. (2009). Biodiversity of exopolysaccharides produced from sucrose by sourdough lactic acid bacteria. J. Agric. Food Chem..

[10] Hu Y., Gänzle M.G. (2018). Effect of temperature on production of oligosaccharides and dextran by *Weissella cibaria* 10 M. Int. J. Food Microbiol..

[11] İspirli H., Özmen D., Yılmaz M.T., Sağdıç O., Dertli E. (2020). Impact of glucan type exopolysaccharide (EPS) production on technological characteristics of sourdough bread. Food Control..

[12] Malang S.K., Maina N.H., Schwab C., Tenkanen M., Lacroix C. (2015). Characterization of exopolysaccharide and ropy capsular polysaccharide formation by Weissella. Food Microbiol..

[13] Manini F., Casiraghi M.C., Poutanen K., Brasca M., Erba D., Plumed-Ferrer C. (2016). Characterization of lactic acid bacteria isolated from wheat bran sourdough. LWT Food Sci. Technol..

[14] Coda R., Xu Y., Moreno D.S., Mojzita D., Nionelli L., Rizzello C.G., Katina K. (2018). Performance of *Leuconostoc citreum* FDR241 during wheat flour sourdough type I propagation and transcriptional analysis of exopolysaccharides biosynthesis genes. Food Microbiol..

[15] Smith J.P., Daifas D.P., El-Khoury W., Koukoutsis J., El-Khoury A. (2004). Shelf life and safety concerns of bakery products—A review. Crit. Rev. Food Sci. Nutr..

[16] Axel C., Röcker B., Brosnan B., Zannini E., Furey A., Coffey A., Arendt E.K. (2015). Application of *Lactobacillus amylovorus* DSM19280 in gluten-free sourdough bread to improve the microbial shelf life. Food Microbiol..

[17] Ryan L.A.M., Zannini E., Dal Bello F., Pawlowska A., Koehler P., Arendt E.K. (2011). *Lactobacillus amylovorus* DSM 19280 as a novel food-grade antifungal agent for bakery products. Int. J. Food Microbiol..

[18] Sadeghi A., Ebrahimi M., Mortazavi S.A., Abedfar A. (2019). Application of the selected antifungal LAB isolate as a protective starter culture in pan whole-wheat sourdough bread. Food Control.

[19] Schmidt M., Lynch K.M., Zannini E., Arendt E.K. (2018). Fundamental study on the improvement of the antifungal activity of *Lactobacillus reuteri* R29 through increased production of phenyllactic acid and reuterin. Food Control.

[20] Dal Bello F., Clarke C.I., Ryan L.A.M., Ulmer H., Schober T.J., Ström K., Sjögren J., van Sinderen D., Schnürer J., Arendt E.K. (2007). Improvement of the quality and shelf life of wheat bread by fermentation with the antifungal strain *Lactobacillus plantarum* FST 1.7. J. Cereal Sci..

[21] Lavermicocca P., Valerio F., Evidente A., Lazzaroni S., Corsetti A., Gobbetti M. (2000). Purification and characteriation of novel antifungal compounds from the sourdough. Appl. Environ. Microbiol..

[22] Black B.A., Zannini E., Curtis J.M., Gänzle M.G. (2013). Antifungal hydroxy fatty acids produced during sourdough fermentation: Microbial and enzymatic pathways, and antifungal activity in bread. Appl. Environ. Microbiol..

[23] Sadiq F.A., Yan B., Tian F., Zhao J., Zhang H., Chen W. (2019). Lactic acid bacteria as antifungal and anti-mycotoxigenic agents: A comprehensive review. Compr. Rev. Food Sci. Food Saf..

[24] Choi H., Kim Y.-W., Hwang I., Kim J., Yoon S. (2012). Evaluation of *Leuconostoc citreum* HO12 and *Weissella koreensis* HO20 isolated from kimchi as a starter culture for whole wheat sourdough. Food Chem..

[25] Ouiddir M., Bettache G., Leyva Salas M., Pawtowski A., Donot C., Brahimi S., Mabrouk K., Coton E., Mounier J. (2019). Selection of Algerian lactic acid bacteria for use as antifungal bioprotective cultures and application in dairy and bakery products. Food Microbiol..

[26] Valerio F., Favilla M., De Bellis P., Sisto A., de Candia S., Lavermicocca P. (2009). Antifungal activity of strains of lactic acid bacteria isolated from a semolina ecosystem against *Penicillium roqueforti*, *Aspergillus niger* and *Endomyces fibuliger* contaminating bakery products. Syst. Appl. Microbiol..

[27] Miescher Schwenninger S., Freimüller Leischtfeld S., Gantenbein-Demarchi C. (2016). high-throughput identification of the microbial biodiversity of cocoa bean fermentation by MALDI-TOF MS. Lett. Appl. Microbiol..

[28] White T.J., Bruns T., Lee S., Taylor J. (1990). Amplification and direct sequencing of fungal ribosomal RNA genes for phylogenetics. PCR Protocols.

[29] Susette F.L., Susanne M.S. (2020). Bread Relevant Moulds. Bak+. Biscuit. Int..

[30] Inglin R.C., Stevens M.J.A., Meile L., Lacroix C., Meile L. (2015). High-throughput screening assays for antibacterial and antifungal activities of Lactobacillus species. J. Microbiol. Methods.

[31] Saha B.C., Nakamura L.K. (2003). Production of mannitol and lactic acid by fermentation with *Lactobacillus intermedius* NRRL B-3693. Biotechnol. Bioeng..

[32] Brosnan B., Coffey A., Arendt E.K., Furey A. (2014). The QuEChERS approach in a novel application for the identification of antifungal compounds produced by lactic acid bacteria Cultures. Talanta.

[33] Müller D.C., Nguyen H., Li Q., Schönlechner R., Miescher Schwenninger S., Wismer W., Gänzle M. (2021). Enzymatic and microbial conversions to achieve sugar reduction in bread. Food Res. Int..

[34] Galli V., Venturi M., Coda R., Maina N.H., Granchi L. (2020). Isolation and characterization of indigenous *Weissella confusa* for in situ bacterial exopolysaccharides (EPS) production in chickpea sourdough. Food Res. Int..

[35] Sheikha A.E., Mahmoud Y.A.-G., Rosell C.M., Bajerska J., El Sheikha A.F. (2015). Bread fungal contamination: Risk of mycotoxins, protection of anti-fungal and need to fungal identification. Bread and Its Fortification.

[36] Mahato D.K., Kamle M., Sharma B., Pandhi S., Devi S., Dhawan K., Selvakumar R., Mishra D., Kumar A., Arora S. (2021). Patulin in food: A Mycotoxin concern for human health and its management strategies. Toxicon.

[37] Le Lay C., Mounier J., Vasseur V., Weill A., Le Blay G., Barbier G., Coton E. (2016). In vitro and in situ screening of lactic acid bacteria and propionibacteria antifungal activities against bakery product Spoilage Molds. Food Control.

[38] Santos J.L.P.d., Bernardi A.O., Pozza Morassi L.L., Silva B.S., Copetti M.V., Sant’Ana A.S. (2016). Incidence, populations and diversity of fungi from raw materials, final products and air of processing environment of multigrain whole meal bread. Food Res. Int..

[39] Samapundo S., Devlieghere F., Vroman A., Eeckhout M. (2017). Antifungal activity of fermentates and their potential to replace propionate in bread. LWT Food Sci. Technol..

[40] Axel C., Brosnan B., Zannini E., Peyer L.C., Furey A., Coffey A., Arendt E.K. (2016). Antifungal activities of three different *Lactobacillus* species and their production of antifungal carboxylic acids in wheat sourdough. Appl. Microbiol. Biotechnol..

[41] Gänzle M.G. (2015). Lactic metabolism revisited: Metabolism of lactic acid bacteria in food fermentations and food spoilage. Curr. Opin. Food Sci..

[42] Zannini E., Waters D.M., Coffey A., Arendt E.K. (2016). Production, properties, and industrial food application of lactic acid bacteria-derived exopolysaccharides. Appl. Microbiol. Biotechnol..

[43] Quattrini M., Liang N., Fortina M.G., Xiang S., Curtis J.M., Gänzle M. (2019). Exploiting synergies of sourdough and antifungal organic acids to delay fungal spoilage of bread. Int. J. Food Microbiol..

[44] Lou X., Si G., Yu H., Qi J., Liu T., Fang Z. (2014). Possible reservoir and routes of transmission of *Cronobacter* (*Enterobacter sakazakii*) via wheat flour. Food Control.

[45] Garófalo L., Vazquez D., Ferreira F., Soule S. (2011). Wheat flour non-starch polysaccharides and their effect on dough rheological properties. Ind. Crop. Prod..

[46] Jung J.-H., Choi N.-Y., Lee S.-Y. (2013). Biofilm formation and exopolysaccharide (EPS) production by *Cronobacter sakazakii* depending on environmental conditions. Food Microbiol..

[47] Almutairi M.H., Helal M.M.I. (2021). Exopolysaccharide production from isolated *Enterobacter* sp. strain ACD2 from the northwest of Saudi Arabia. J. King Saud Univ. Sci..

